# Risk factors and healthcare costs associated with long bone fracture non-union: a retrospective US claims database analysis

**DOI:** 10.1186/s13018-023-04232-3

**Published:** 2023-10-03

**Authors:** Mari F. Vanderkarr, Jill W. Ruppenkamp, Mollie Vanderkarr, Chantal E. Holy, Michael Blauth

**Affiliations:** 1 MedTech Epidemiology, Johnson & Johnson, New Brunswick, NJ USA; 2 DePuy Synthes, West Chester, PA USA; 3 DePuy Synthes, Zuchwil, SO Switzerland; 4 Somerville, USA

**Keywords:** Fractures, Non-union, Risk factors, Bone fractures, Cost analysis

## Abstract

**Background:**

Few contemporary US-based long bone non-union analyses have recently been published. Our study was designed to provide a current understanding of non-union risks and costs, from the payers' perspective.

**Methods:**

The Merative™ MarketScan^®^ Commercial Claims and Encounters database was used. Patients with surgically treated long bone (femur, tibia, or humerus) fractures in the inpatient setting, from Q4 2015 to most recent, were identified. Exclusion criteria included polytrauma and amputation at index. The primary outcome was a diagnosis of non-union in the 12 and 24 months post-index. Additional outcomes included concurrent infection, reoperation, and total healthcare costs. Age, gender, comorbidities, fracture characteristics, and severity were identified for all patients. Descriptive analyses were performed. Crude and adjusted rates of non-union (using Poisson regressions with log link) were calculated. Marginal incremental cost of care associated with non-union and infected non-union and reoperation were estimated using a generalized linear model with log link and gamma distribution.

**Results:**

A total of 12,770, 13,504, and 4,805 patients with femoral, tibial, or humeral surgically treated fractures were identified, 74–89% were displaced, and 18–27% were comminuted. Two-year rates of non-union reached 8.5% (8.0%–9.1%), 9.1% (8.6%–9.7%), and 7.2% (6.4%–8.1%) in the femoral, tibial, and humeral fracture cohorts, respectively. Shaft fractures were at increased risk of non-union versus fractures in other sites (risk ratio (RR) in shaft fractures of the femur: 2.36 (1.81–3.04); tibia: 1.95 (1.47–2.57); humerus: 2.02 (1.42–2.87)). Fractures with severe soft tissue trauma (open vs. closed, Gustilo III vs. Gustilo I–II) were also at increased risk for non-union (RR for Gustilo III fracture (vs. closed) for femur: R = 1.96 (1.45–2.58), for tibia: 3.33 (2.85–3.87), RR for open (vs. closed) for humerus: 1.74 (1.30–2.32)). For all fractures, younger patients had a reduced risk of non-union compared to older patients. For tibial fractures, increasing comorbidity (Elixhauser Index 5 or greater) was associated with an increased risk of non-union. The two-year marginal cost of non-union ranged from $33K-$45K. Non-union reoperation added $16K–$34K in incremental costs. Concurrent infection further increased costs by $46K–$86K.

**Conclusions:**

Non-union affects 7–10% of surgically treated long bone fracture cases. Shaft and complex fractures were at increased risk for non-union.

**Supplementary Information:**

The online version contains supplementary material available at 10.1186/s13018-023-04232-3.

## Background

Long bone fractures are common in the United States (US), with proximal femur fractures affecting as many as 294 cases per 100,000 person-years [1]. The economic impact of these fractures is considerable. A 2013 study estimated the costs of long bone fractures in working-age US patients at approximately $23K per affected limb [2]. More recently, these estimates were increased to $35K per fracture site, in Medicare patients [3].

Non-union represents one of the main complications of fracture healing. A 2007 study reported non-union rates as high as 12% in patients with femoral fracture requiring external fixation and 80% in patients with Gustilo III tibial fractures [4]. A more recent, UK-based analysis estimated overall non-union risks, across all long bone fractures, at 1.9%, with rates as high as 9% for some age groups and fracture anatomies [5].

The costs associated with non-union further increase the economic burden associated with long bone fractures. Patients with non-union were shown in a 2013 study to incur a median all-cause, post-fracture cost of care of $25,556 versus $11,686 for patients without non-union [6].

Risk factors for non-union have been reported in multiple studies, with limited concordance between studies. A 2016 prediction model of tibial non-union reported increased risk based on variables such as use of flaps, development of compartmental syndrome, overall chronic conditions, American Society of Anesthesiologist (ASA) score and male gender [7]. Other studies have identified male gender, smoking, diabetes and poor surgical fixation of the fracture site, as potential risk factors [8, 9].

Recently, costs and risk factors for non-union were evaluated using the Victorian Orthopaedic Trauma Outcomes Registry (VOTOR) in Australia. This study identified an overall risk of non-union across the entire registry of 8%, resulting in an incremental cost of care of AUD $4.9M. Risk factors for non-union included age and fracture type, with highest risks being identified for patients with femoral or tibial shaft fractures. Interestingly, patients receiving compensation (social insurance) due to their injuries were also at increased risk for non-union [10].

To the best of our knowledge, recent, detailed, risk and cost analyses of non-union following long bone fractures in the US have not been reported. Our study was therefore designed to evaluate, in a large US claims database, the risk and direct healthcare costs—from the perspective of commercial payers—of non-union complications, following femoral, tibial or humeral fractures that necessitate surgical intervention.

## Methods

### Data

The Merative™ MarketScan^®^ Commercial Claims and Encounters (CCAE) database was used for this analysis. The CCAE database represents data from individuals enrolled in US employer-sponsored insurance health plans. Approximately 138 million enrollees are included in this database. The data include adjudicated health insurance claims (e.g., inpatient, outpatient, and outpatient pharmacy) as well as enrollment data from large employers and health plans who provide private healthcare coverage to employees, their spouses and dependents. Additionally, it captures laboratory tests for a subset of the covered lives. The major data elements contained within this database are outpatient pharmacy dispensing claims (coded with National Drug Codes (NDC)), inpatient and outpatient medical claims which provide procedure codes (coded in CPT-4, HCPCS, ICD-9-PCS or ICD-10-PCS) and diagnosis codes (coded in ICD-9-CM or ICD-10-CM).

*Institutional Review Board (IRB):* The data used in this analysis were de-identified and cannot be re-identified. As such, the use of the MarketScan^﻿®^ data is exempt from broad IRB approval.

*Cohort:* Adult and pediatric patients with a first diagnosis of long bone (femur, tibia or humerus) fracture in the inpatient setting, and a surgical orthopedic fracture repair procedure within 3 months of first fracture diagnosis, from October 1st, 2015, to December 31, 2021, were identified. The inpatient admission with fracture repair surgery was identified as the index, and the admission date for this admission, as the index date. All patients had at least 30 days of continuous enrollment after index. Exclusion criteria included: more than one fracture type, amputation at index, less than 6 months of pre-index medical history in the database, prior fractures in the 6 months pre-index period and diagnoses indicative of prior fracture complications (i.e., non-union, malunion, infection, delayed healing) in the 6 months pre-index.

### Outcomes

The primary outcome was a diagnosis of non-union in the 12 and 24 months post-index. Additional outcomes included concurrent diagnoses of infection, reoperations with non-union diagnoses and all-cause reoperations, and total all-cause cost of care from index to 12 and 24 months post-index.

### Variables

Age, gender and comorbidities defined using the Elixhauser comorbidity index (ECI) [11] and all 31 chronic diseases identified by the ECI were identified for all patients, based on 6-month pre-index medical history. The ECI was selected because this metric was shown in prior research to have strong predictability for morbidity and mortality risk, especially in orthopedic surgery cases [12, 13]. Variables specific to fracture anatomy (e.g., shaft fracture, condylar fracture), severity (e.g., closed vs. Gustilo Type I-II vs. Gustilo Type III) and treatment and fixation type were also captured for all patients. The fracture anatomy categories for patients with femoral fractures included trochanteric fractures, femoral neck fractures, shaft fractures, condylar fractures, fractures at multiple sites within the femur and unspecified/other fractures. For tibial fractures, categories included condylar fractures, other proximal fractures, shaft fractures, pilon and malleolus fractures, other distal fractures, fractures at multiple sites within the tibia and unspecified/other fractures. For humerus fractures, categories were as follows: neck fractures, tuberosity fractures, other proximal fractures, shaft fractures, condyle fractures, other distal fractures, fractures at multiple sites within the humerus and unspecified/other fractures.

### Analyses

Three distinct cohorts of patients were analyzed separately: patients with femoral, tibial and humeral fracture. As stated above in the exclusion criteria, no patient had concurrent fractures of two or more long bone types. For each cohort, all study variables were analyzed descriptively. Counts and proportions were provided for dichotomous and polychotomous variables. Measures of central tendency and spread were calculated for continuous variables (e.g., mean and standard deviation (SD) or median and IQR, depending on variable distribution). Rates of non-union, with and without fracture-related infection and with and without reoperations, for each cohort, were determined using Kaplan-Meier survival analyses. Adjusted rates, and two-year risk factors for non-union, were modeled using Poisson regressions with log link. A time-varying covariate was added to the Poisson regression, to adjust for the fact that not all patients had 2-year post-index continuous enrollment in the database. Marginal incremental cost of care associated with non-union, with and without infection and reoperation, was defined as the marginal incremental all-cause cost of care at 12 and 24 months between patients with no complications vs. non-union, infection and reoperation, and were estimated using a generalized linear model (GLM) with log link and gamma distribution. For cost estimates, only patients with complete 12-month follow-up (for 12-month costs) and 24-month follow-up (for 24-month costs) were included in the analyses. All costs are shown as mean with 95% confidence intervals. All analyses were performed in R version 4.1.3 [2022-03-10].

## Results

### Cohort

The cohort size, demographics, comorbidities and fracture characteristics for each fracture anatomy are shown in Table 1. The cohort of patients with femoral, tibial and humeral fracture requiring surgical repair included 12,770, 13,504 and 4805 patients, respectively. For all 3 cohorts, the average age ranged from 36 to 44, with more than a third of all patients in the 55–64 age category. Patients with femur fracture had the highest ECI (1.61, SD: 2.20) compared to patients with tibial (1.04, SD: 1.60) or humeral fractures (1.16, SD: 1.77). Patients with femur fracture were more likely to have 5 or more comorbidities (10.2%) versus patients with fractures of the tibia (4.4%) or the humerus (6.0%). Diabetes and hypertension were common comorbidities. Diabetes affected between 10-14% of patients, whereas hypertension was reported in 22–28% of patients, and 8–9% of patients were obese across all 3 cohorts. Shaft fractures affected 37.9% (femur), 57.1% (tibia) and 40.2% (humerus) cases, and half of all femur fractures were located in the femoral neck (50.3%). For each long bone, demographic and clinical characteristics of patients were further stratified by the exact anatomy of the fracture within the long bone (e.g., for femur: trochanteric, neck, shaft or condylar). Tables for each long bone are shown in the Additional file 1: Tables S1, S2 and S3. These tables provide demographic, comorbid and treatment type (intramedullary nailing, internal fixation and external fixation) for each fracture anatomy. More than 74% of all fractures were displaced and 18–27% were comminuted. These findings are expected since all cases had surgical fracture repair. For femur fractures, 2.7% were Gustilo Type III versus 10% of tibial fractures and < 1% of humerus fractures. Fixation type was different across cohorts; approximately one half of femur fractures were treated with intramedullary nails (48.0%), whereas tibial and humeral fractures were mostly treated with internal fixation (53.5% and 75.7%, respectively).

**Table 1:** Demographic, clinical and fracture characteristics of patients with fractures requiring surgical repair

	Femur	Tibia	Humerus
***N***	12,770	13,504	4,805
**Gender: Male versus Female**	6,435 (50.4%)	7,026 (52.0%)	2,159 (44.9%)
**Age (Mean (SD))**	44.17 (19.46)	42.21 (16.03)	36.26 (22.19)
**Age group**			
Under 19	2,089 (16.4%)	1,548 (11.5%)	1,477 (30.7%)
19–25	1,174 (9.2%)	1,417 (10.5%)	391 (8.1%)
26–34	667 (5.2%)	1,386 (10.3%)	253 (5.3%)
35–44	861 (6.7%)	2,068 (15.3%)	390 (8.1%)
45–54	1,910 (15.0%)	3,017 (22.3%)	698 (14.5%)
55–64	6,069 (47.5%)	4,068 (30.1%)	1,596 (33.2%)
**Elixhauser index (Mean (SD))**	1.61 (2.20)	1.04 (1.60)	1.16 (1.77)
**Elixhauser category**			
0: No comorbidities	5,713 (44.7%)	7,259 (53.8%)	2,587 (53.8%)
1 or 2	3,921 (30.7%)	4,393 (32.5%)	1,394 (29.0%)
3 or 4	1,832 (14.3%)	1,261 (9.3%)	534 (11.1%)
5 or greater	1,304 (10.2%)	591 (4.4%)	290 (6.0%)
**Comorbidities**			
Diabetes	1,739 (13.6%)	1,304 (9.7%)	504 (10.5%)
Hypertension	3,587 (28.1%)	2,950 (21.8%)	1,091 (22.7%)
Depression	1,741 (13.6%)	1,645 (12.2%)	555 (11.6%)
Chronic pulmonary diseases	1,324 (10.4%)	952 (7.0%)	405 (8.4%)
Fluid and electrolyte disorders	1,114 (8.7%)	488 (3.6%)	258 (5.4%)
Hypothyroidism	1,124 (8.8%)	892 (6.6%)	360 (7.5%)
Cardiac arrythmia	1,043 (8.2%)	657 (4.9%)	278 (5.8%)
Obesity	982 (7.7%)	1,196 (8.9%)	402 (8.4%)
Neurological disorders	739 (5.8%)	349 (2.6%)	192 (4.0%)
Metastatic cancer	687 (5.4%)	69 (0.5%)	120 (2.5%)
Deficiency anemia	609 (4.8%)	301 (2.2%)	112 (2.3%)
Rheumatoid arthritis	554 (4.3%)	332 (2.5%)	141 (2.9%)
**Fracture type**			
Trochanteric fracture^a^	4,607 (36.1%)	NA	N/A
Neck fracture^b^	6,417 (50.3%)	NA	844 (17.6%)
Shaft fracture	4,844 (37.9%)	7,715 (57.1%)	1,933 (40.2%)
Condylar fracture	1,120 (8.8%)	3,709 (27.5%)	1,858 (38.7%)
Other / unspecified	987 (7.7%)	162 (1.2%)	82 (1.7%)
Pilon / malleolus fracture^c^	N/A	2,832 (21.0%)	N/A
Proximal end fracture^d^	N/A	2,643 (19.6%)	1,662 (34.6%)
Distal end fracture^d^	N/A	2,770 (20.5%)	1,614 (33.6%)
Tuberosity fracture^e^	N/A	NA	399 (8.3%)
**Clinical presentation**			
Displaced fracture	9,531 (74.6%)	12,054 (89.3%)	3,938 (82.0%)
Comminuted fracture	2,299 (18.0%)	3,509 (26.0%)	1,283 (26.7%)
**Fracture Gustilo**			
Closed	11,088 (86.8%)	9,857 (73.0%)	4,027 (83.8%)
Open type I or II	1,034 (8.1%)	2,268 (16.8%)	697 (14.5%)
Open type III	351 (2.7%)	1,315 (9.7%)	12 (0.2%)
Unknown	297 (2.3%)	64 (0.5%)	69 (1.4%)
**Fixation type**			
Internal fixation	4,987 (39.1%)	7,226 (53.5%)	3,638 (75.7%)
Intramedullary fixation	6,127 (48.0%)	3,635 (26.9%)	304 (6.3%)
External fixation	193 (1.5%)	2,466 (18.3%)	110 (2.3%)

^a^Femur only

^b^Femur and humerus only

^c^Tibia only

^d^Tibia and Humerus only. Proximal and distal end fractures include physeal, Salter-Harris, and torus fractures, as well as cases defined as "upper end" or "lower end" fractures

^e^Humerus only

### Risk of non-union

**Fig. 1:**
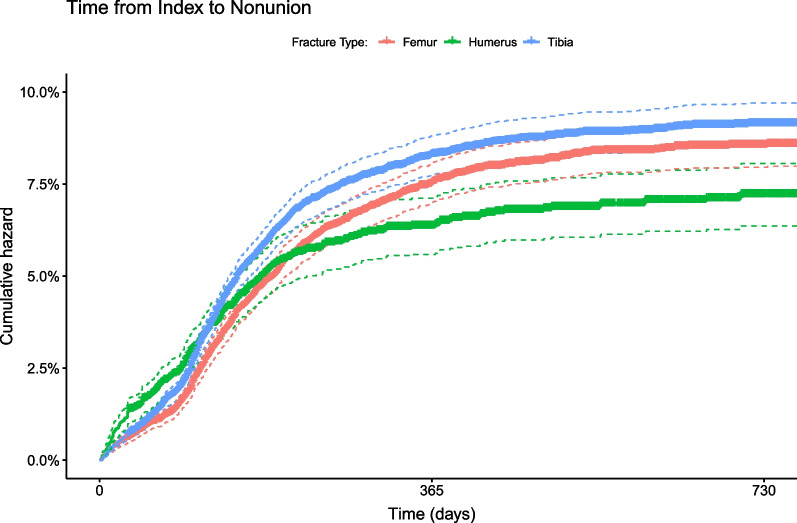
Cumulative hazard for non-union, from day of discharge following surgical fracture repair, to 2 years post-surgery. Cumulative hazard for non-union following surgical repair of femoral, tibial and humeral fracture reached 8.5% (8.0%–9.1%), 9.1% (8.6%–9.7%) and 7.2% (6.4%–8.1%), respectively

The cumulative hazards for non-union following surgical repair of long bone fractures are shown in Fig. 1**,**, and the actual 12- and 24-month rates of non-union are presented in Table 2. As seen in Fig. 1, patients with tibial fractures were at increased risk for non-union compared to patients with femoral or humeral fractures, and most non-union cases were identified by 12-month follow-up. Rates of non-union at 12 and 24 months post-surgery were 8.3% (7.8%–8.8%) and 9.1% (8.6%–9.7%) for patients with tibial fractures, 7.5% (7.0%–8.0%) and 8.5% (8.0%–9.1%) for patients with femoral fractures and 6.4% (5.6%–7.1%) and 7.2% (6.4%–8.1%) for patients with humeral fractures. In all cases, infected non-unions were uncommon, less than 1% for femoral and humeral fractures, and approximately 2% for tibial fractures presented with infected non-unions. Not all patients with non-union had immediate reoperation. All patients with tibial non-union were reoperated by the 2-year follow-up period; however, only 6% of the 8.5% of patients with femoral non-unions (70.1%), and only 4.9% of the 7.2% of patients with the humeral non-unions (68.1%) were reoperated by 2 years post-index.

**Table 2:** Rates of non-union, non-union with infection and reoperations for non-union, at 365- and 730-day follow-up

	Femur		Tibia		Humerus	
	365-day	730-day	365-day	730-day	365-day	730-day
All non-union cases	7.5% (7.0%–8.0%)	8.5% (8.0%–9.1%)	8.3% (7.8%–8.8%)	9.1% (8.6%–9.7%)	6.4% (5.6%–7.1%)	7.2% (6.4%–8.1%)
Infected non-union	0.6% (0.4%–0.7%)	0.6% (0.4%–0.7%)	1.9% (1.7%–2.2%)	2.0% (1.7%–2.3%)	0.9% (0.6%–1.2%)	0.9% (0.6%–1.2%)
Reoperated non-union*	4.7% (4.3%–5.1%)	6.0% (5.5%–6.5%)	7.0% (6.5%–7.4%)	9.1% (8.5%–9.7%)	3.8% (3.2%–4.4%)	4.9% (4.2%–5.7%)

^*^By end of follow-up period

### Risks and risk ratios in patients with femoral fractures requiring surgery

**Fig. 2:**
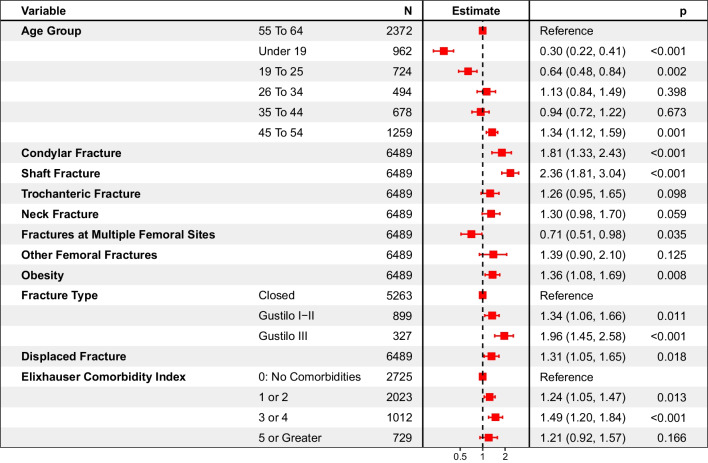
Forest plot of 2-year risk ratios for non-union following femoral fractures requiring surgery

The cumulative hazard of non-union was analyzed separately for each femoral fracture anatomy, as shown in Additional file 1: Fig. S1, as significant differences in hazard were observed. Excluding patients with undefined femoral fracture anatomy, or fractures at multiple femoral sites, the cumulative hazard at 2 years post-index for non-union in patients with condylar, shaft, trochanteric or neck fractures was 9.8% (7.0%–12.6%), 9.0% (7.8%–10.2%), 6.9% (5.3%–8.5%) and 6.0% (4.9%–7.0%), respectively. Two-year risk ratios (RR) for non-union following femoral fractures are shown in Fig. 2. The adjusted rate of non-union for patients in the 55–64 age group (majority age group), with no comorbidities, no obesity and fractures that were closed, non-displaced, without distal end or shaft involvement was 2.1% (95% CI: 1.5%–3.0%). The impact of each of these different variables on the risk of non-union is shown in Fig. 2. As expected, the highest increase in risk for non-union was observed in patients with shaft involvement (RR: 2.36 (95% CI: 1.81–3.04)), followed by patients with condylar fractures (RR: 1.81 (95% CI: 1.33–2.43)). Gustilo Type III fractures were also at significantly increased risk (RR: 1.96 (95% CI: 1.45–2.58)) compared to patients with closed fractures.

### Risks and risk ratios in patients with tibial fractures requiring surgery

**Fig. 3:**
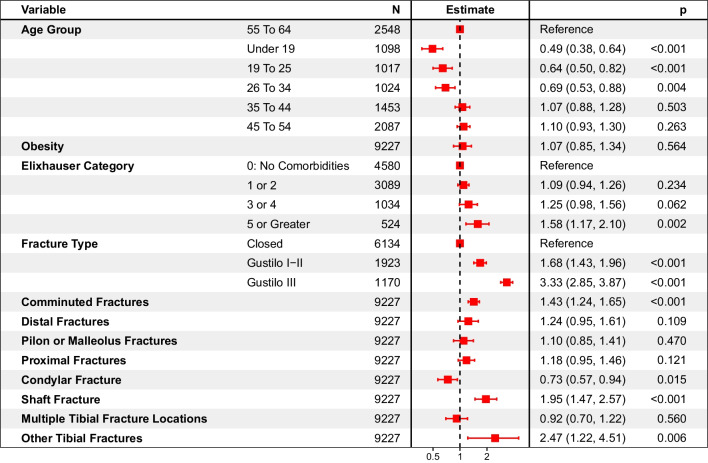
Forest plot of 2-year risk ratios for non-union following tibial fractures requiring surgery

The Additional file 1: Fig. S2 shows cumulative hazard for non-union for each tibial fracture anatomy. Excluding patients with undefined tibial fracture anatomy, or fractures at multiple tibial sites, the cumulative hazard at 2 years post-index for non-union in patients with shaft, distal, pilon or malleolus, condyle and proximal fracture was 10.8% (9.2%–12.3%), 8.1% (4.7%–11.4%), 5.2% (4.3%–6.1%), 2.4% (1.5%–3.3%) and 2.2% (0.0%–4.6%), respectively. The adjusted rate of non-union for patients in the 55–64 age group, with no comorbidities, no obesity and fractures that were closed, non-comminuted, without distal end or shaft involvement was 2.6% (95% CI: 1.9%–3.5%). The impact of each of the different variables on the risk of non-union is shown in Fig. 3. Patients with comminuted and shaft fracture were at increased risk (Comminuted: RR: 1.43 (95% CI: 1.24–1.65) − Shaft: RR: 1.95 (95% CI: 1.47–2.57)). Gustilo Type I-II and III fractures (vs. closed) were associated with a significant increase in risk for non-union (Gustilo Type I-II: RR: 1.68 (95% CI: 1.43–1.96) − Gustilo Type III: RR: 3.33 (95% CI: 2.85–3.87)). Concurrent fractures within the tibia also resulted in significantly greater risks of non-union (RR: 2.47 (95% CI: 1.22–4.51)). Unlike femoral fractures, for patients with tibial fractures, having 5 or more comorbidities increased risk of non-union (RR: 1.58 (95% CI: 1.17–2.10)).

### Risks and risk ratios in patients with humeral fractures requiring surgery

The Additional file 1: Fig. S3 shows cumulative hazard for non-union for each humeral fracture anatomy. Excluding patients with undefined humeral fracture anatomy, or fractures at multiple humeral sites, the cumulative hazard at 2 years post-index for non-union in patients with shaft, proximal, neck, distal, tuberosity and condylar fracture was 14.9% (11.5%–18.2%), 6.9% (3.7%–9.9%), 6.3% (0.0%–13.0%), 3.3% (1.2%–5.4%), 2.9% (0.0%–8.2%) and 0.5% (0.0%–1.1%), respectively.

**Fig. 4:**
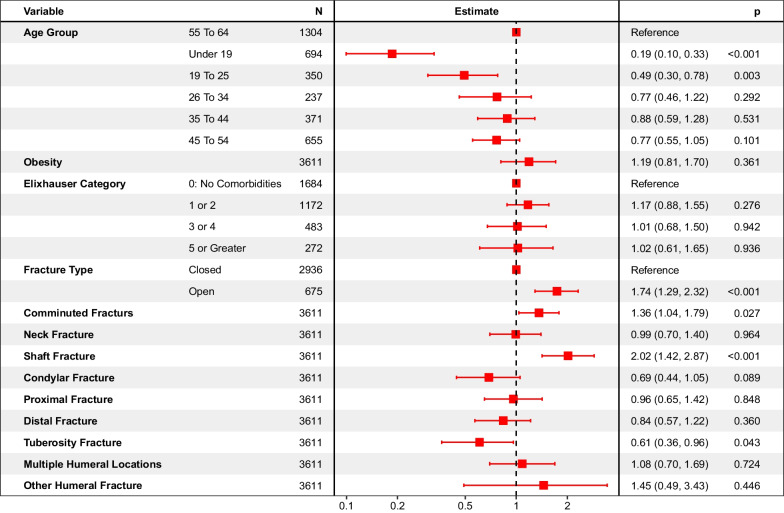
Forest plot of 2-year risk ratios for non-union following humeral fractures requiring surgery

The adjusted rate of non-union for patients in the 55–64 age group, with no comorbidities, no obesity and fractures that are closed, non-comminuted, without shaft involvement was 3.9% (95% CI: 2.5%—6.1%). As observed following femoral or tibial fracture, patients with a fracture of the humeral shaft were at increased risk of non-union compared to patients that did not have a humeral shaft fracture (RR: 2.02 (95% CI: 1.42–2.87)). Only a few Gustilo III cases were available, the analysis was thus conducted comparing patients with open versus closed fracture, regardless of Gustilo categorization. Patients with open fracture were at increased risk of non-union (RR: 1.74 (95% CI: 1.29–2.32)), and patients with comminuted fractures were also at a slight increased risk (RR: 1.36 (95% CI: 1.04–1.79)), as shown in Fig. 4**.** Patient comorbidities did not significantly impact risk of non-union.

### Cost of non-union

The cost to commercial payers for non-union cases is shown in Table 3, along with cost of concurrent fracture-related infection and reoperations. Non-union by itself, without reoperation or infection, resulted in an increased marginal cost of care in patients with femoral, tibial and humeral fracture at 2 years post-index of $45,633 ($23,913–$67,352), $40,409 ($30,295–$50,523) and $33,308 ($14,603– $52,013), respectively. Reoperations added between $16K and $34K of incremental costs. When infection was also present, costs increased by an additional $46K–$86K. Femoral complications were more expensive than tibial or humeral complications, and humeral complications had the lowest incremental costs (vs. femoral or tibial).

**Table 3:** Incremental cost of care of patients with non-union, non-union with infection and reoperations

		365-day follow-up	730-day follow-up
Femur	Incremental payer cost of non-union Additional cost for:	$27,982 ($17,816–$38,148)	$45,633 ($23,913–$67,352)
	Deep Infection	$63,908 ($42,404–$85,413)	$86,983 ($42,399–$131,566)
	Reoperation for non-union	$22,865 ($15,671–$30,060)	$25,941 ($11,914–$39,969)
Tibia	Incremental payer cost of non-union Additional cost for:	$31,502 ($25,818–$37,185)	$40,409 ($30,295–$50,523)
	Deep Infection	$52,016 ($43,456–$60,576)	$71,235 ($56,363–$86,107)
	Reoperation for non-union	$33,550 ($29,588–$37,511)	$34,327 ($27,543–$41,112)
Humerus	Incremental payer cost of non-union Additional cost for:	$20,925 ($9,101–$32,748)	$33,308 ($14,603–$52,013)
	Deep Infection	$34,580 ($11,815–$57,345)	$46,325 ($12,189–$80,461)
	Reoperation for non-union	$16,775 ($9,484–$24,067)	$16,183 ($4,723–$27,643)

The incremental cost for non-union represents the added cost to payers when patients presented with a diagnosis of non-union. When patients developed non-union with deep infection, they incurred additional costs, shown separately in the table above. Similarly, when patients underwent a reoperation for their non-union, the additional costs for the procedures are shown separately from the cost of non-union and/or deep infection. The total costs for patients with infected, reoperated non-union are therefore the sum of the costs of non-union, deep infection and reoperation, as shown in this table

## Discussion

We analyzed contemporary rates of non-union in the United States (US) in patients presenting with long bone fractures requiring surgical care, aged less than 65. Two-year rates of non-union across the entire cohort following femoral, tibial and humeral fractures with surgery reached 8.5% (8.0%–9.1%), 9.1% (8.6%–9.7%) and 7.2% (6.4%–8.1%), respectively. Adjusted risks of non-union, for patients with closed fractures and no comorbidities, ranged from 2% to 4%. Presence of fractures with severe soft tissue trauma increased those risks significantly, the greatest increase in risk was observed for tibial Gustilo III fractures, which came with a risk ratio of 3.3 (95% CI: 2.85–3.87) vs closed tibial fracture. Marginal cost of non-union was significant, ranging from $33K–$45K at 2 years post-index. When patients had undergone a reoperation, those costs further increased by $16K–$34K, and a concurrent fracture-related infection diagnosis further increased those costs by $46K–$86K.

Most non-union cases were identified in our study between 3 months and 365 days post-operative. This timeframe is expected based on the definition of non-union, described as complications of fractures with no signs of healing for 3 months and persistence for a minimum of 9 months [14]. At 12 months, between 80% and 90% cases of non-union were diagnosed, as shown in Table 2. This finding was consistent with prior reports on time to non-union [15, 16].

Our observed rates of non-union between 7.2% and 9.1% at 2 years are comparable to those reported by a recent Australian registry study, which described an overall 8% non-union rate. [10] A difference between our study and the Australian registry study, is that we only included patients that underwent surgical treatment for their fractures, whereas the Australian registry study included all patients with an inpatient admission > 24 h for fracture care, regardless of surgical intervention. The reason for including only patients with surgical intervention in our study, was to exclude patients with minimal risk for any treatment or complications, for example patients with closed or stress fractures, which may resolve with limited medical intervention. It is likely that these patients were also excluded from the Australian registry analysis, as they may not have required the > 24 h inpatient admission. Another US-based claims database analysis looking at non-unions in 2011 reported rates for tibia and femur fractures at 14% and 13.9%, respectively. These findings are higher than those identified in our study and may reflect inclusion of patients with polytrauma, which were excluded in our study [17].

Specifically for femoral shaft fractures, two recent studies have described non-union rates ranging from 5.8% for patients from a single Level 1 trauma center treated with intramedullary nails [18] to 15.8% for patients from multiple sites with segmental shaft fractures [19]. In our study, femoral shaft fractures had a risk of non-union of 9.0% (7.8%–10.2%). The differences in these reported rates may be related to the different inclusion/exclusion criteria used for each study. In contrast to these two studies, our study included all patients with femoral shaft fractures requiring surgical intervention in the claims database, from 2015 to 2021, with a primary fracture diagnosis, no amputation at index, and at least 6 months of medical history pre-index. Our cohort thus included a sizable proportion of patients with severe fracture (47.4% comminuted fractures, as shown in Table S1), with the majority treated by intramedullary nail, but also external fixators.

We observed a delay between diagnoses of non-union and reoperation. At the 1-year post-operative time point, a third of humeral non-union and half of femoral and tibial non-union had been reoperated. This delay is expected, as a non-union diagnosis may take time to be confirmed. Because of this delay, we reported costs of reoperation separately.

At 2 years post-index, concurrent fracture-related infection was reported in a small minority of non-union cases. The significant increase in cost due to the presence of infection is intuitive and has been reported by others. Patients with septic non-union have been shown to undergo much more extensive care, for longer duration, compared to patients with aseptic non-union [20].

Our study has the following limitations: As with all database research, the data used for these analyses were not specifically designed for this study. We relied on presence of diagnostic and procedural codes, to define variables and outcomes. Coding errors or missing codes present in the databases, despite validations performed by MarketScan^®^, would not be identifiable as such. In addition, the exact type of non-union pathologies, and the detailed procedural approaches used to address these complications, were not analyzed. Key details such as the type of hospital that treated the patients (e.g., Level 1 Trauma centers versus community hospitals) were also not available. The exact causes leading to complications are also unknown based on the database analyses, thus preventing us from using the findings from our study for clinical recommendations. An additional limitation may be related to the generalizability of the findings: our study used a large claims database of insured patients, who may be socioeconomically favored compared to patients with no insurance or Medicaid insurance coverage. Our study also did not include race and ethnicity information, which may impact the generalizability of the findings to minority groups. A key strength of our research is the use of contemporary data, thus reflecting the latest standard of care, and the size of the available population, for statistical analyses.

From a clinical guideline standpoint, our study highlights the fact that non-union represents a significant complication that cannot be overlooked. Whereas the severity of fractures (e.g., Gustilo type) impacts risk of non-union, patient comorbidities, especially multiple concurrent comorbidities as shown with the Elixhauser Index, significantly increase those risks. Whereas our study was not designed to identify the exact surgical factors (e.g., fixation types or surgical techniques) associated with non-union, our findings do suggest that patients that have any of the risk factors listed herein should be monitored closely for potential non-union.

Whereas risk factors for non-union are starting to be understood, there is still a large amount of uncertainty in terms of who may suffer from non-union. Future research should focus on studies where detailed patient records (including activity levels and social determinants of health) and clinical factors (including detailed surgical technique variables, device types, hospital types and provider coordination) are captured, thus allowing further understanding and reduction of non-union risks. These insights may require prospective studies and/or data analyses from large registries containing detailed patient and surgical data.

## Conclusions

Two-year non-union rates following surgically treated femoral, tibial and humeral fracture reached 8.5% (8.0%–9.1%), 9.1% (8.6%–9.7%) and 7.2% (6.4%–8.1%), respectively. Risk for non-union significantly increased in shaft fractures, and fractures with severe soft tissue trauma (Gustilo III / open fractures). Adjusted risks of non-union, for patients with closed fractures, no comorbidities and low-severity fractures (not comminuted or displaced), ranged from 2% to 4%. The incremental cost of care in patients with non-union ranged from $33K to $45K, prior to cost of reoperation. This cost increased by an average of $16K–$34K for non-union reoperation and by $46K–$86K in patients with concurrent fracture-related infection.

### Supplementary Information

**Additional file 1:** Tables detailing demographic and clinical characteristics of patients for, and figures displaying cumulative hazard for non-union following surgical repair of, each long bone separately.

## Data Availability

The data for these analyses were made available to the authors by third-party licenses from Merative™ MarketScan^®^, a data provider in the US. Under the licensing agreement, the authors cannot provide raw data themselves. Other researchers could access the data by purchase through MarketScan﻿^®^, and the inclusion criteria specified in the Methods section would allow them to identify the same cohort of patients we used for these analyses.

## Abbreviations

ASA: American Society of Anesthesiologist physical status classification system
CI: Confidence interval
ECI: Elixhauser comorbidity index
GLM: Generalized linear model
RR: Risk ratio
SD: Standard deviation
VOTOR: Victorian Orthopedic Trauma Outcomes Registry
