# Supplementary material for: Risk factors and healthcare costs associated with long bone fracture non-union: a retrospective US claims database analysis
Source: J Orthop Surg Res. 2023 Oct 3;18:745. doi: 10.1186/s13018-023-04232-3 (PMC10546674; doi:10.1186/s13018-023-04232-3)
Supplement: Supplementary file 1 — Additional file 1: Tables detailing demographic and clinical characteristics of patients for, and figures displaying cumulative hazard for non-union following surgical repair of, each long bone separately. [file 13018_2023_4232_MOESM1_ESM.docx]

**Figure S1:** Cumulative hazard over 2 years for non-union following femur fracture, by fracture anatomy. Excluding patients with undefined femoral fracture anatomy, or fractures at multiple femoral sites, the cumulative hazard at 2 years post-index for nonunion in patients with condylar, shaft, trochanteric or neck fractures was 9.8% (7.0%-12.6%), 9.0% (7.8%-10.2%), 6.9% (5.3%-8.5%) and 6.0% (4.9%-7.0%), respectively.

**
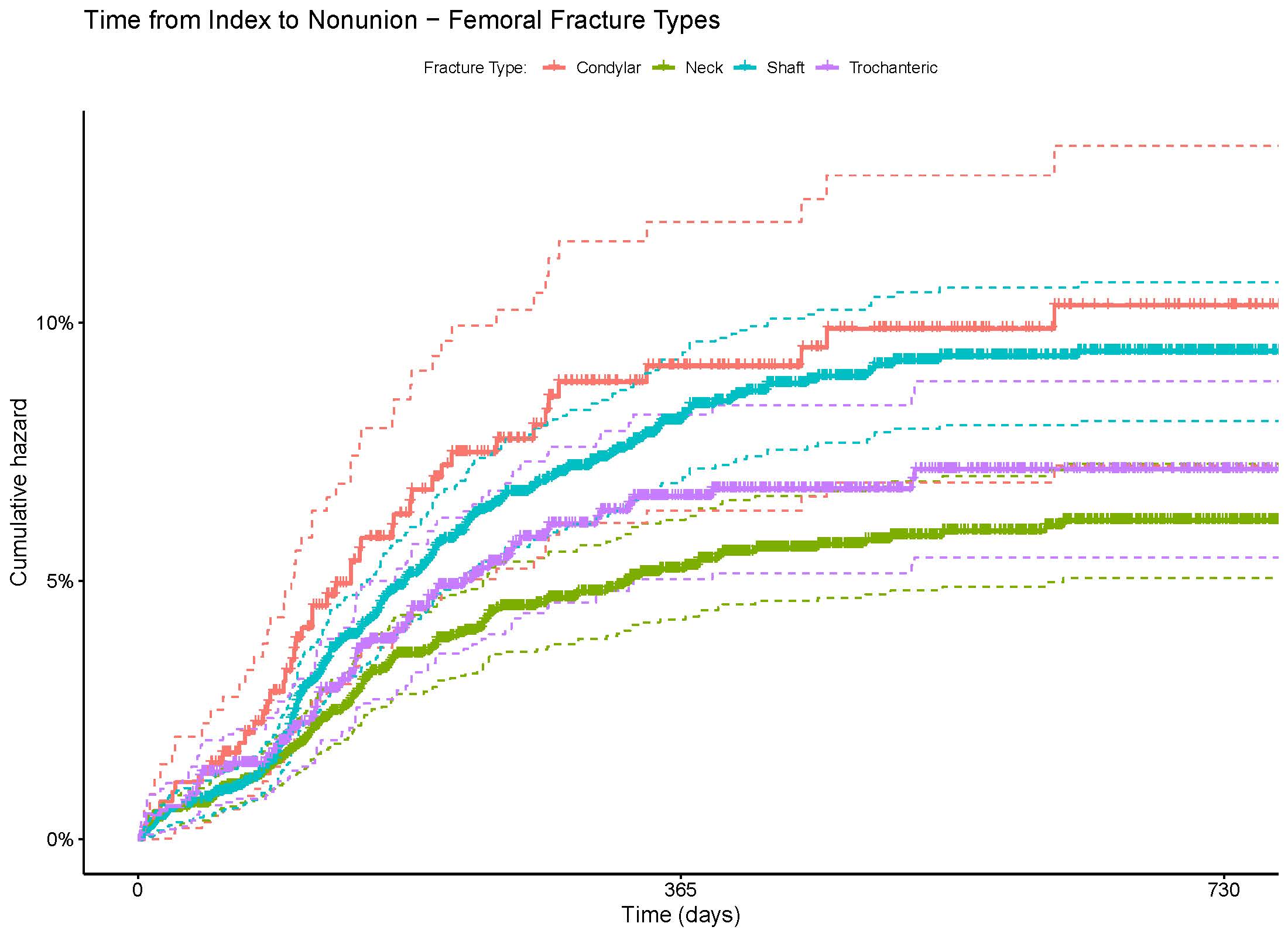
**

**Figure S2:** Cumulative hazard over 2 years for non-union following tibial fracture, by fracture anatomy. Excluding patients with undefined tibial fracture anatomy or fracture at multiple tibial sites, the cumulative hazard at 2 years post-index for nonunion in patients with shaft, distal, pilon or malleolus, condyle and proximal fracture was 10.8% (9.2%-12.3%), 8.1% (4.7%-11.4%), 5.2% (4.3%-6.1%), 2.4% (1.5%-3.3%) and 2.2% (0.0%-4.6%), respectively.


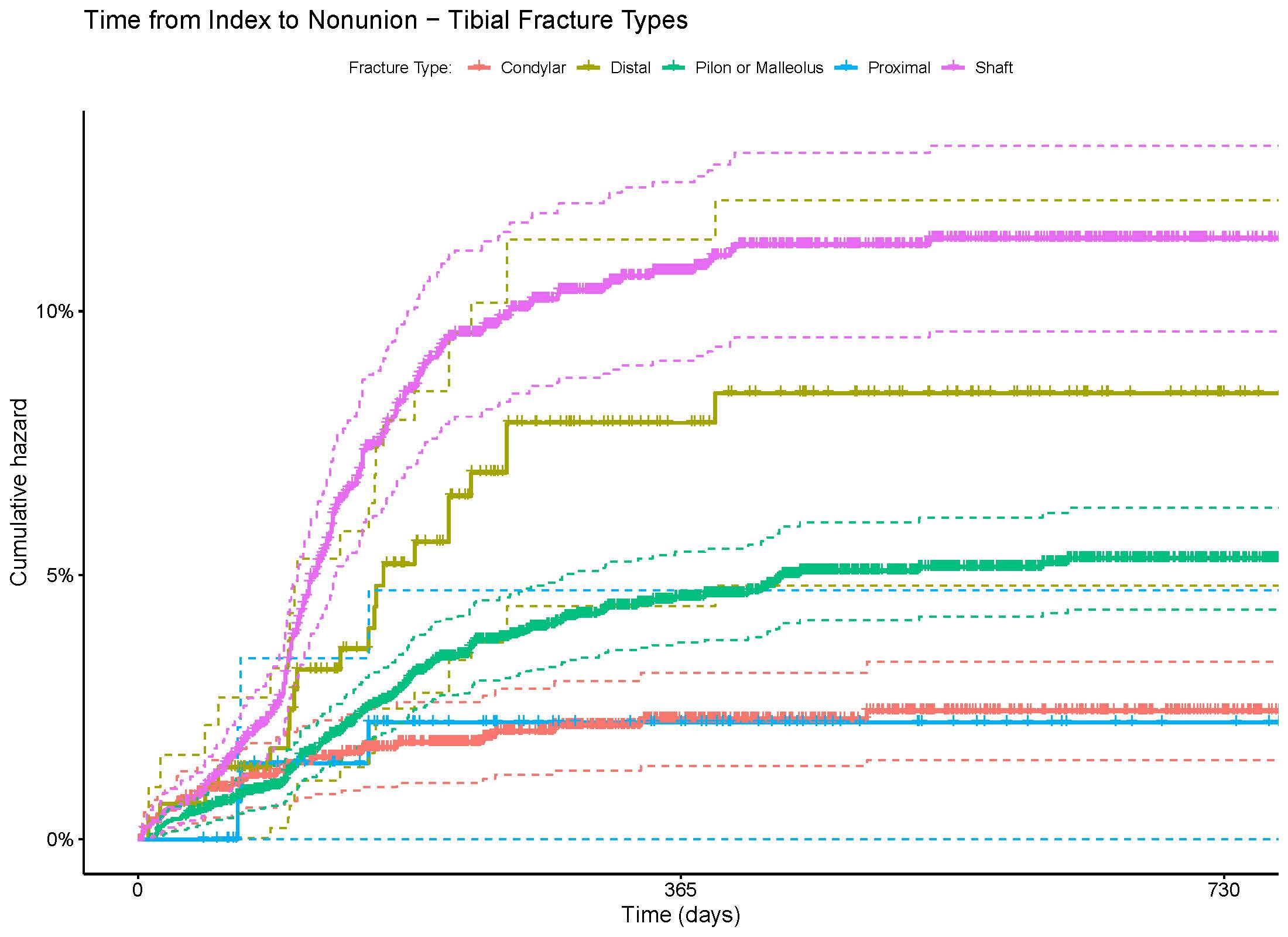


**Figure S3:** Cumulative hazard over 2 years for non-union following humeral fracture, by fracture anatomy. Excluding patients with undefined humeral fracture anatomy or fracture at multiple humeral sites, the cumulative hazard at 2 years post-index for nonunion in patients with shaft, proximal, neck, distal, tuberosity and condylar fracture was 14.9% (11.5%-18.2%), 6.9% (3.7%-9.9%) ,6.3% (0.0%-13.0%), 3.3% (1.2%-5.4%), 2.9% (0.0%-8.2%) and 0.5% (0.0%-1.1%), respectively.
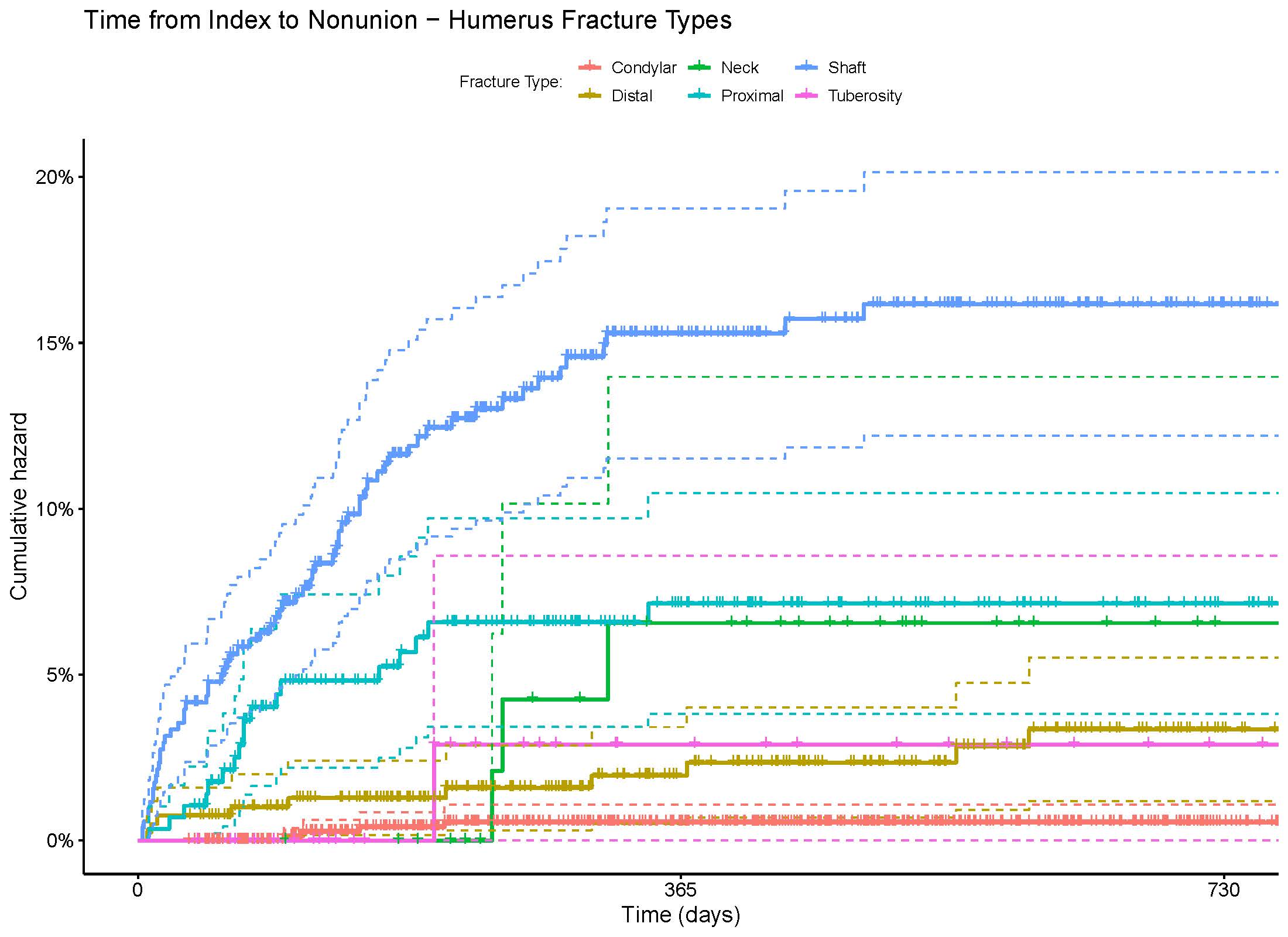


**Table S1:** Demographic, comorbid and clinical presentation of patients with femoral fracture, by femoral anatomy

| **Fracture anatomy:** | Trochanteric | Neck | Shaft | Condylar | Other/  Unspecified | Multiple |
| --- | --- | --- | --- | --- | --- | --- |
| **N** | 1,252 | 2,665 | 2,664 | 547 | 987 | 4,655 |
| **Gender: Male (vs Female)** | 598 (47.8%) | 1,047 (39.3%) | 1,745 (65.5%) | 240 (43.9%) | 477 (48.3%) | 2,328 (50.0%) |
| **Age (Mean (SD))** | 52.70 (13.66) | 51.87 (13.86) | 27.45 (19.25) | 45.46 (18.02) | 43.47 (20.07) | 47.02 (17.93) |
| **Age Group** |  |  |  |  |  |  |
| under 19 | 56 (4.5%) | 153 (5.7%) | 1,063 (39.9%) | 70 (12.8%) | 224 (22.7%) | 523 (11.2%) |
| 19 to 25 | 50 (4.0%) | 85 (3.2%) | 536 (20.1%) | 54 (9.9%) | 44 (4.5%) | 405 (8.7%) |
| 26 to 34 | 42 (3.4%) | 91 (3.4%) | 223 (8.4%) | 39 (7.1%) | 29 (2.9%) | 243 (5.2%) |
| 35 to 44 | 84 (6.7%) | 196 (7.4%) | 189 (7.1%) | 35 (6.4%) | 67 (6.8%) | 290 (6.2%) |
| 45 to 54 | 200 (16.0%) | 519 (19.5%) | 221 (8.3%) | 82 (15.0%) | 169 (17.1%) | 719 (15.4%) |
| 55 to 64 | 820 (65.5%) | 1,621 (60.8%) | 432 (16.2%) | 267 (48.8%) | 454 (46.0%) | 2,475 (53.2%) |
| **Elixhauser Index (Mean (SD))** | 1.96 (2.20) | 2.00 (2.52) | 0.70 (1.39) | 1.59 (2.07) | 2.14 (2.29) | 1.69 (2.23) |
| **Elixhauser Category** |  |  |  |  |  |  |
| 0: No comorbidities | 407 (32.5%) | 987 (37.0%) | 1,797 (67.5%) | 237 (43.3%) | 321 (32.5%) | 1,964 (42.2%) |
| 1 or 2 | 446 (35.6%) | 886 (33.2%) | 619 (23.2%) | 174 (31.8%) | 315 (31.9%) | 1,481 (31.8%) |
| 3 or 4 | 262 (20.9%) | 419 (15.7%) | 159 (6.0%) | 85 (15.5%) | 195 (19.8%) | 712 (15.3%) |
| 5 or greater | 137 (10.9%) | 373 (14.0%) | 89 (3.3%) | 51 (9.3%) | 156 (15.8%) | 498 (10.7%) |
| **Clinical Presentation** |  |  |  |  |  |  |
| Displaced Fracture | 1,185 (94.6%) | 949 (35.6%) | 2,362 (88.7%) | 518 (94.7%) | 62 (6.3%) | 4,455 (95.7%) |
| Comminuted Fracture | 0 (0.0%) | 0 (0.0%) | 1,262 (47.4%) | 0 (0.0%) | 0 (0.0%) | 1,037 (22.3%) |
| **Fracture Gustilo** |  |  |  |  |  |  |
| Closed | 1,192 (95.2%) | 2,550 (95.7%) | 2,263 (84.9%) | 413 (75.5%) | 554 (56.1%) | 4,116 (88.4%) |
| Open Type I or II | 52 (4.2%) | 99 (3.7%) | 277 (10.4%) | 97 (17.7%) | 123 (12.5%) | 386 (8.3%) |
| Open Type III | 8 (0.6%) | 16 (0.6%) | 124 (4.7%) | 37 (6.8%) | 13 (1.3%) | 153 (3.3%) |
| Unknown | 0 (0.0%) | 0 (0.0%) | 0 (0.0%) | 0 (0.0%) | 297 (30.1%) | 0 (0.0%) |
| **Fixation Type** |  |  |  |  |  |  |
| Internal Fixation | 348 (27.8%) | 1,646 (61.8%) | 566 (21.2%) | 405 (74.0%) | 428 (43.4%) | 1,594 (34.2%) |
| Intramedullary Fixation | 829 (66.2%) | 182 (6.8%) | 1,846 (69.3%) | 38 (6.9%) | 273 (27.7%) | 2,959 (63.6%) |
| External Fixation | 4 (0.3%) | 22 (0.8%) | 58 (2.2%) | 29 (5.3%) | 10 (1.0%) | 70 (1.5%) |

**Table S2:** Demographic, comorbid and clinical presentation of patients with tibial fracture, by tibial anatomy

| **Fracture anatomy:** | Condylar | Other Proximal | Shaft | Pilon and Malleolus | Other Distal | Other/  Unspecified | Multiple |
| --- | --- | --- | --- | --- | --- | --- | --- |
| **N** | 1,258 | 146 | 1,692 | 2,832 | 299 | 162 | 7,115 |
| **Gender: Male (vs Female)** | 581 (46.2%) | 107 (73.3%) | 1,119 (66.1%) | 1,100 (38.8%) | 177 (59.2%) | 98 (60.5%) | 3,844 (54.0%) |
| **Age (Mean (SD))** | 47.31 (13.20) | 30.49 (20.18) | 33.64 (16.48) | 45.16 (14.70) | 36.13 (18.91) | 31.68 (20.96) | 42.91 (15.49) |
| **Age Group** |  |  |  |  |  |  |  |
| under 19 | 36 (2.9%) | 77 (52.7%) | 405 (23.9%) | 158 (5.6%) | 91 (30.4%) | 86 (53.1%) | 695 (9.8%) |
| 19 to 25 | 93 (7.4%) | 9 (6.2%) | 323 (19.1%) | 277 (9.8%) | 23 (7.7%) | 5 (3.1%) | 687 (9.7%) |
| 26 to 34 | 105 (8.3%) | 5 (3.4%) | 210 (12.4%) | 302 (10.7%) | 33 (11.0%) | 6 (3.7%) | 725 (10.2%) |
| 35 to 44 | 202 (16.1%) | 5 (3.4%) | 222 (13.1%) | 433 (15.3%) | 19 (6.4%) | 5 (3.1%) | 1,182 (16.6%) |
| 45 to 54 | 338 (26.9%) | 15 (10.3%) | 257 (15.2%) | 657 (23.2%) | 61 (20.4%) | 18 (11.1%) | 1,671 (23.5%) |
| 55 to 64 | 484 (38.5%) | 35 (24.0%) | 275 (16.3%) | 1,005 (35.5%) | 72 (24.1%) | 42 (25.9%) | 2,155 (30.3%) |
| **Elixhauser Index (Mean (SD))** | 1.15 (1.56) | 1.10 (1.73) | 0.65 (1.20) | 1.25 (1.79) | 1.02 (1.52) | 1.42 (2.13) | 1.01 (1.58) |
| **Elixhauser Category** |  |  |  |  |  |  |  |
| 0: No comorbidities | 597 (47.5%) | 85 (58.2%) | 1,123 (66.4%) | 1,375 (48.6%) | 162 (54.2%) | 78 (48.1%) | 3,839 (54.0%) |
| 1 or 2 | 459 (36.5%) | 36 (24.7%) | 439 (25.9%) | 961 (33.9%) | 97 (32.4%) | 53 (32.7%) | 2,348 (33.0%) |
| 3 or 4 | 151 (12.0%) | 16 (11.0%) | 99 (5.9%) | 326 (11.5%) | 28 (9.4%) | 16 (9.9%) | 625 (8.8%) |
| 5 or greater | 51 (4.1%) | 9 (6.2%) | 31 (1.8%) | 170 (6.0%) | 12 (4.0%) | 15 (9.3%) | 303 (4.3%) |
| **Clinical Presentation** |  |  |  |  |  |  |  |
| Displaced Fracture | 1,247 (99.1%) | 51 (34.9%) | 1,349 (79.7%) | 2,809 (99.2%) | 5 (1.7%) | 77 (47.5%) | 6,516 (91.6%) |
| Comminuted Fracture | 0 (0.0%) | 0 (0.0%) | 812 (48.0%) | 0 (0.0%) | 0 (0.0%) | 0 (0.0%) | 2,697 (37.9%) |
| **Fracture Gustilo** |  |  |  |  |  |  |  |
| Closed | 1,128 (89.7%) | 119 (81.5%) | 1,112 (65.7%) | 2,163 (76.4%) | 220 (73.6%) | 89 (54.9%) | 5,026 (70.6%) |
| Open Type I or II | 112 (8.9%) | 14 (9.6%) | 377 (22.3%) | 443 (15.6%) | 49 (16.4%) | 8 (4.9%) | 1,265 (17.8%) |
| Open Type III | 18 (1.4%) | 13 (8.9%) | 203 (12.0%) | 226 (8.0%) | 30 (10.0%) | 1 (0.6%) | 824 (11.6%) |
| Unknown | 0 (0.0%) | 0 (0.0%) | 0 (0.0%) | 0 (0.0%) | 0 (0.0%) | 64 (39.5%) | 0 (0.0%) |
| **Fixation Type** |  |  |  |  |  |  |  |
| Internal Fixation | 1,040 (82.7%) | 84 (57.5%) | 326 (19.3%) | 1,957 (69.1%) | 184 (61.5%) | 90 (55.6%) | 3,545 (49.8%) |
| Intramedullary Fixation | 4 (0.3%) | 7 (4.8%) | 1,222 (72.2%) | 12 (0.4%) | 51 (17.1%) | 20 (12.3%) | 2,319 (32.6%) |
| External Fixation | 174 (13.8%) | 4 (2.7%) | 103 (6.1%) | 597 (21.1%) | 40 (13.4%) | 4 (2.5%) | 1,544 (21.7%) |

**Table S3:** Demographic, comorbid and clinical presentation of patients with humeral fracture, by humeral anatomy

| **Fracture anatomy:** | Neck | Tuberosity | Other Proximal | Shaft | Other Distal | Condyle | Other/ Unspecified | Multiple |
| --- | --- | --- | --- | --- | --- | --- | --- | --- |
| **N** | 56 | 47 | 288 | 516 | 401 | 823 | 82 | 2,592 |
| **Gender: Male (vs Female)** | 22 (39.3%) | 29 (61.7%) | 102 (35.4%) | 239 (46.3%) | 184 (45.9%) | 424 (51.5%) | 40 (48.8%) | 1,119 (43.2%) |
| **Age (Mean (SD))** | 54.68 (11.23) | 45.26 (15.16) | 51.77 (13.27) | 41.34 (17.02) | 45.43 (16.85) | 10.09 (12.42) | 49.90 (15.88) | 39.43 (21.00) |
| **Age Group** |  |  |  |  |  |  |  |  |
| under 19 | 2 (3.6%) | 1 (2.1%) | 14 (4.9%) | 58 (11.2%) | 41 (10.2%) | 748 (90.9%) | 6 (7.3%) | 607 (23.4%) |
| 19 to 25 | 1 (1.8%) | 8 (17.0%) | 8 (2.8%) | 86 (16.7%) | 40 (10.0%) | 9 (1.1%) | 5 (6.1%) | 234 (9.0%) |
| 26 to 34 | 1 (1.8%) | 5 (10.6%) | 13 (4.5%) | 52 (10.1%) | 26 (6.5%) | 10 (1.2%) | 2 (2.4%) | 144 (5.6%) |
| 35 to 44 | 2 (3.6%) | 5 (10.6%) | 24 (8.3%) | 63 (12.2%) | 40 (10.0%) | 13 (1.6%) | 7 (8.5%) | 236 (9.1%) |
| 45 to 54 | 12 (21.4%) | 11 (23.4%) | 62 (21.5%) | 88 (17.1%) | 74 (18.5%) | 17 (2.1%) | 17 (20.7%) | 417 (16.1%) |
| 55 to 64 | 38 (67.9%) | 17 (36.2%) | 167 (58.0%) | 169 (32.8%) | 180 (44.9%) | 26 (3.2%) | 45 (54.9%) | 954 (36.8%) |
| **Elixhauser Index (Mean (SD))** | 1.61 (1.74) | 1.77 (1.87) | 2.02 (2.03) | 1.30 (1.75) | 1.16 (1.63) | 0.24 (0.78) | 3.29 (2.26) | 1.25 (1.83) |
| **Elixhauser Category** |  |  |  |  |  |  |  |  |
| 0: No comorbidities | 17 (30.4%) | 16 (34.0%) | 85 (29.5%) | 241 (46.7%) | 189 (47.1%) | 706 (85.8%) | 9 (11.0%) | 1,324 (51.1%) |
| 1 or 2 | 26 (46.4%) | 18 (38.3%) | 103 (35.8%) | 183 (35.5%) | 152 (37.9%) | 99 (12.0%) | 25 (30.5%) | 788 (30.4%) |
| 3 or 4 | 9 (16.1%) | 8 (17.0%) | 66 (22.9%) | 51 (9.9%) | 46 (11.5%) | 13 (1.6%) | 21 (25.6%) | 320 (12.3%) |
| 5 or greater | 4 (7.1%) | 5 (10.6%) | 34 (11.8%) | 41 (7.9%) | 14 (3.5%) | 5 (0.6%) | 27 (32.9%) | 160 (6.2%) |
| **Clinical Presentation** |  |  |  |  |  |  |  |  |
| Displaced Fracture | 21 (37.5%) | 47 (100.0%) | 167 (58.0%) | 389 (75.4%) | 74 (18.5%) | 820 (99.6%) | 0 (0.0%) | 2,420 (93.4%) |
| Comminuted Fracture | 0 (0.0%) | 0 (0.0%) | 0 (0.0%) | 226 (43.8%) | 0 (0.0%) | 176 (21.4%) | 0 (0.0%) | 881 (34.0%) |
| **Fracture Gustilo** |  |  |  |  |  |  |  |  |
| Closed | 54 (96.4%) | 46 (97.9%) | 270 (93.8%) | 429 (83.1%) | 301 (75.1%) | 771 (93.7%) | 18 (22.0%) | 2,138 (82.5%) |
| Open Type I or II | 2 (3.6%) | 1 (2.1%) | 16 (5.6%) | 86 (16.7%) | 98 (24.4%) | 50 (6.1%) | 0 (0.0%) | 444 (17.1%) |
| Open Type III | 0 (0.0%) | 0 (0.0%) | 0 (0.0%) | 0 (0.0%) | 1 (0.2%) | 1 (0.1%) | 0 (0.0%) | 10 (0.4%) |
| Unknown | 0 (0.0%) | 0 (0.0%) | 2 (0.7%) | 1 (0.2%) | 1 (0.2%) | 1 (0.1%) | 64 (78.0%) | 0 (0.0%) |
| **Fixation Type** |  |  |  |  |  |  |  |  |
| Internal Fixation | 38 (67.9%) | 31 (66.0%) | 179 (62.2%) | 335 (64.9%) | 306 (76.3%) | 709 (86.1%) | 15 (18.3%) | 2,025 (78.1%) |
| Intramedullary Fixation | 1 (1.8%) | 0 (0.0%) | 18 (6.3%) | 77 (14.9%) | 4 (1.0%) | 15 (1.8%) | 35 (42.7%) | 154 (5.9%) |
| External Fixation | 0 (0.0%) | 1 (2.1%) | 3 (1.0%) | 18 (3.5%) | 23 (5.7%) | 13 (1.6%) | 0 (0.0%) | 52 (2.0%) |
